# EMBL’s European Bioinformatics Institute (EMBL-EBI) in 2024

**DOI:** 10.1093/nar/gkae1089

**Published:** 2024-11-28

**Authors:** Matthew Thakur, Catherine Brooksbank, Robert D Finn, Helen V Firth, Julia Foreman, Mallory Freeberg, Kim T Gurwitz, Melissa Harrison, David Hulcoop, Sarah E Hunt, Andrew R. Leach, Mariia Levchenko, Diana Marques, Ellen M McDonagh, Aziz Mithani, Helen Parkinson, Yasset Perez-Riverol, Zinaida Perova, Ugis Sarkans, Santosh Tirunagari, Eleni Tzampatzopoulou, Aravind Venkatesan, Juan-Antonio Vizcaino, Benjamin Wingfield, Barbara Zdrazil, Johanna McEntyre

**Affiliations:** European Molecular Biology Laboratory, European Bioinformatics Institute (EMBL-EBI), Wellcome Genome Campus, Hinxton, CB10 1SA, UK; European Molecular Biology Laboratory, European Bioinformatics Institute (EMBL-EBI), Wellcome Genome Campus, Hinxton, CB10 1SA, UK; European Molecular Biology Laboratory, European Bioinformatics Institute (EMBL-EBI), Wellcome Genome Campus, Hinxton, CB10 1SA, UK; Wellcome Sanger Institute, Wellcome Genome Campus, Hinxton, CB10 1SA, UK; Cambridge University Hospitals NHS Foundation Trust, East Anglian Medical Genetics Service, Hills Road, Cambridge, CB2 0QQ, UK; European Molecular Biology Laboratory, European Bioinformatics Institute (EMBL-EBI), Wellcome Genome Campus, Hinxton, CB10 1SA, UK; European Molecular Biology Laboratory, European Bioinformatics Institute (EMBL-EBI), Wellcome Genome Campus, Hinxton, CB10 1SA, UK; European Molecular Biology Laboratory, European Bioinformatics Institute (EMBL-EBI), Wellcome Genome Campus, Hinxton, CB10 1SA, UK; European Molecular Biology Laboratory, European Bioinformatics Institute (EMBL-EBI), Wellcome Genome Campus, Hinxton, CB10 1SA, UK; Wellcome Sanger Institute, Wellcome Genome Campus, Hinxton, CB10 1SA, UK; Open Targets, Wellcome Genome Campus, Hinxton, CB10 1SA, UK; European Molecular Biology Laboratory, European Bioinformatics Institute (EMBL-EBI), Wellcome Genome Campus, Hinxton, CB10 1SA, UK; European Molecular Biology Laboratory, European Bioinformatics Institute (EMBL-EBI), Wellcome Genome Campus, Hinxton, CB10 1SA, UK; European Molecular Biology Laboratory, European Bioinformatics Institute (EMBL-EBI), Wellcome Genome Campus, Hinxton, CB10 1SA, UK; European Molecular Biology Laboratory, European Bioinformatics Institute (EMBL-EBI), Wellcome Genome Campus, Hinxton, CB10 1SA, UK; Wellcome Sanger Institute, Wellcome Genome Campus, Hinxton, CB10 1SA, UK; Open Targets, Wellcome Genome Campus, Hinxton, CB10 1SA, UK; European Molecular Biology Laboratory, European Bioinformatics Institute (EMBL-EBI), Wellcome Genome Campus, Hinxton, CB10 1SA, UK; European Molecular Biology Laboratory, European Bioinformatics Institute (EMBL-EBI), Wellcome Genome Campus, Hinxton, CB10 1SA, UK; European Molecular Biology Laboratory, European Bioinformatics Institute (EMBL-EBI), Wellcome Genome Campus, Hinxton, CB10 1SA, UK; European Molecular Biology Laboratory, European Bioinformatics Institute (EMBL-EBI), Wellcome Genome Campus, Hinxton, CB10 1SA, UK; European Molecular Biology Laboratory, European Bioinformatics Institute (EMBL-EBI), Wellcome Genome Campus, Hinxton, CB10 1SA, UK; European Molecular Biology Laboratory, European Bioinformatics Institute (EMBL-EBI), Wellcome Genome Campus, Hinxton, CB10 1SA, UK; European Molecular Biology Laboratory, European Bioinformatics Institute (EMBL-EBI), Wellcome Genome Campus, Hinxton, CB10 1SA, UK; European Molecular Biology Laboratory, European Bioinformatics Institute (EMBL-EBI), Wellcome Genome Campus, Hinxton, CB10 1SA, UK; European Molecular Biology Laboratory, European Bioinformatics Institute (EMBL-EBI), Wellcome Genome Campus, Hinxton, CB10 1SA, UK; European Molecular Biology Laboratory, European Bioinformatics Institute (EMBL-EBI), Wellcome Genome Campus, Hinxton, CB10 1SA, UK; European Molecular Biology Laboratory, European Bioinformatics Institute (EMBL-EBI), Wellcome Genome Campus, Hinxton, CB10 1SA, UK; European Molecular Biology Laboratory, European Bioinformatics Institute (EMBL-EBI), Wellcome Genome Campus, Hinxton, CB10 1SA, UK

## Abstract

The European Molecular Biology Laboratory’s European Bioinformatics Institute (EMBL-EBI) is one of the world’s leading sources of public biomolecular data. Based at the Wellcome Genome Campus in Hinxton, UK, EMBL-EBI is one of six sites of the European Molecular Biology Laboratory, Europe’s only intergovernmental life sciences organization. This overview summarizes the latest developments in services that EMBL-EBI data resources provide to scientific communities globally (https://www.ebi.ac.uk/services).

## Graphical Abstract

**Graphical Abstract:**
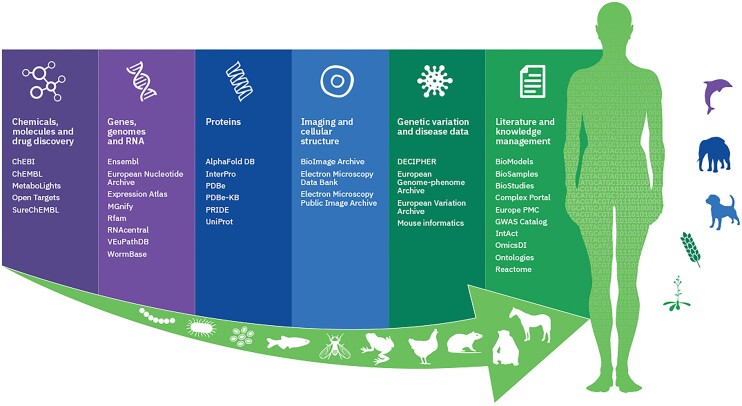

## Introduction

The European Molecular Biology Laboratory’s European Bioinformatics Institute (EMBL-EBI) is one of the world’s leading sources of public biomolecular data. Based at the Wellcome Genome Campus in Hinxton, UK, EMBL-EBI is one of six sites of the European Molecular Biology Laboratory, Europe’s only intergovernmental life sciences organization. EMBL-EBI’s vision is to benefit humankind by advancing scientific discovery and impact through bioinformatics. To achieve this, EMBL-EBI collaborates with scientists, clinicians and engineers all over the world to provide the infrastructure and tools necessary to share life science data openly.

This overview focuses on services that EMBL-EBI data resources provide to scientific communities globally, and associated training activities. Many other EMBL-EBI data resources have dedicated articles in this special issue—this overview summarizes major changes to the data resources not described elsewhere.

EMBL-EBI data resources accessed via the EMBL-EBI services web portal comprise deposition databases, which archive experimental data; knowledgebases, which provide annotation, curation, reanalysis and integration of deposited data; and open source software tools that enable reuse of these resources. All EMBL-EBI data resources and many software systems can be downloaded and installed locally, and our licensing strategy is to make resources available on an open and free basis for reuse wherever possible with ‘no additional restriction on the use of the contributed data than those specified by the data owner’. EMBL-EBI data services offer further bulk and machine-readable access including via API, FTP, Google BigQuery, Aspera and Globus services.

EMBL-EBI data resources serve as foundations for hundreds of downstream external resources, research programmes and tools, including as input to large language models (LLMs). An overarching trend across web-based resources is rising user adoption of natural language, LLM-based query interfaces. These interfaces are now able to retrieve results relating to EMBL-EBI data resources in real time. Internal work and external collaborations refining and applying LLM (see below) are enabling us to understand current limitations and optimize resources for this emerging user interface.

### The impact of EMBL-EBI data resources

EMBL-EBI monitors the overall use of data resources including the volume of data deposited to the archival resources, as well as the number of web requests and unique IP addresses visiting the data resource websites. While each metric has limitations, considered together they give an indication of the scale and trend of usage.

**Figure 1.**
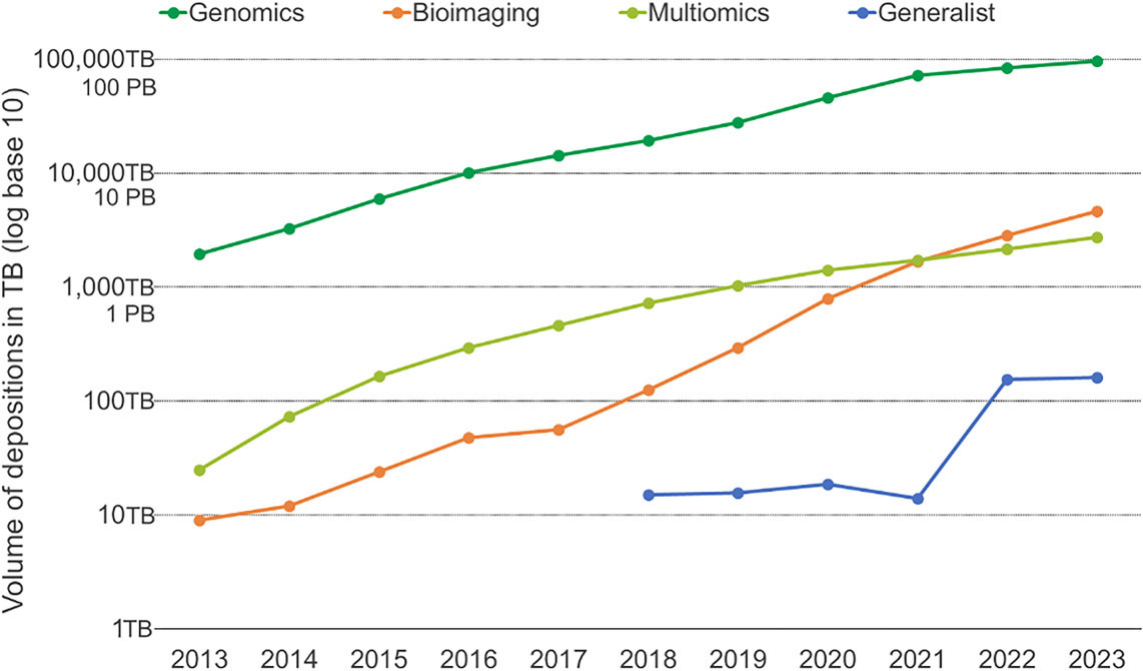
Cumulative volumes of data depositions into EMBL-EBI archival data resources in Terabytes. Note the log scale (base 10). Genomics includes ENA, EGA and European Variation Archive. Bioimaging includes EMPIRE, BIA and EMDB. Multiomics includes PRIDE and MetaboLights. Generalist includes BioStudies.

The rate of data depositions by volume into EMBL-EBI’s archival resources continues to accelerate, with over 15 Petabytes of data deposited in 2023, bringing the cumulative user depositions to ∼105 Petabytes (Figure 1, below). The largest archival resources are genomics-focussed—European Nucleotide Archive (ENA) (1) and European Genome-phenome Archive (EGA) (2), accounting for over 91% of total data deposited to date. In recent years, imaging data resources have seen rapid growth, namely, the BioImage Archive (BIA) (3); and the electron microscopy imaging resources Electron Microscopy Public Image Archive (4) and Electron Microscopy Data Bank (EMDB) (5).

**Figure 2.**
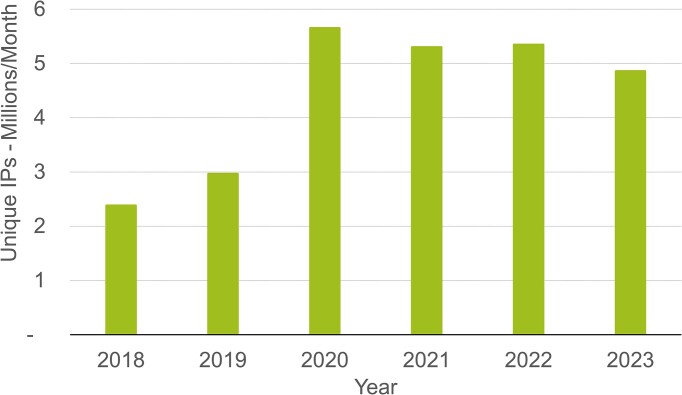
Monthly average unique IP addresses visiting EMBL-EBI data resources between 2018 and 2023.

After an increase in demand that coincided with the COVID-19 pandemic in 2020, usage has remained high and in 2023 an average of 4.8 million unique IP addresses (Figure 2) generated 3 billion web requests per month. This is over 100% more unique IP addresses accessing EMBL-EBI data resources than in 2018. EMBL-EBI data use is truly global, with every UN member state country represented in our user base.

## New developments in deposition databases

### Calculating polygenic scores in the context of genetic ancestry with the PGS Catalog Calculator

The PGS Catalog (6) is the world’s largest FAIR (Findable, Accessible, Interoperable and Reusable) repository of polygenic scores (PGSs) and relevant metadata required to evaluate and reuse them. A PGS is a measurement which represents genetic predisposition for a heritable trait or phenotype. Widespread use of cohorts with predominantly European ancestry has resulted in many PGSs performing poorly in individuals with non-European ancestry.

For example, the mean and variance of calculated PGS distributions can differ across genetic ancestry groups due to differing linkage disequilibrium patterns and allelic frequencies with true change in risk (e.g. in biomarker values). Incorporating genetic ancestry information when calculating PGS mitigates this statistical artefact and allows comparison of relative risk. Although the PGS Catalog provides formatted and standardized data, calculating and adjusting PGSs across ancestries previously required different software tools and considerable bioinformatics and statistics expertise.

The PGS Catalog Calculator is a portable and reproducible workflow which automates PGS calculation, genetic ancestry similarity analysis and PGS adjustment. The calculator requires imputed target genotypes in VCF or PLINK format and scoring files (i.e. a list of variants with effect alleles and their associated weights, available in the PGS Catalog) to calculate PGSs, which are returned to the user in a text file with an accompanying summary report. Deep integration with the PGS Catalog API simplifies the process of identifying and using multiple scoring files in the correct genome build.

**Figure 3.**
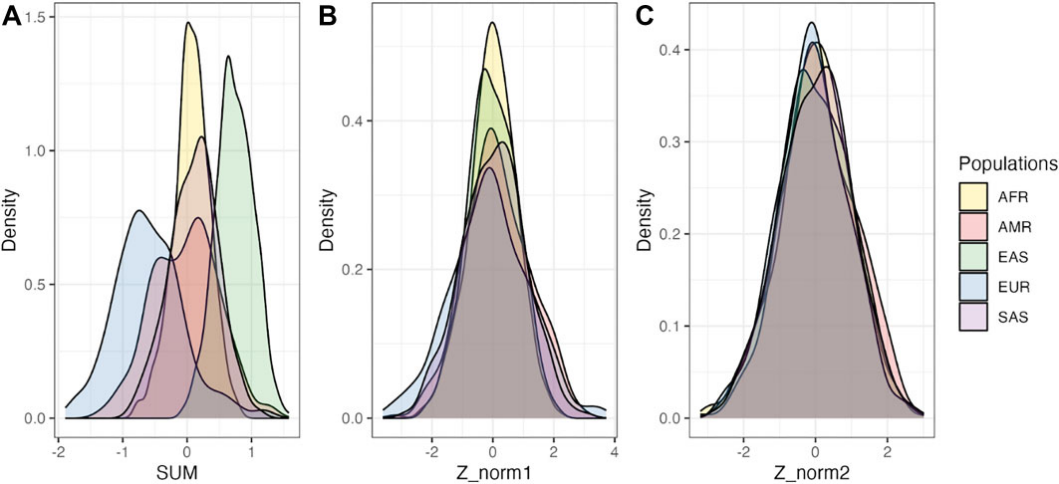
Density plots of PGSs show PGSs are confounded by genetic ancestry. A PGS is the weighted sum of effect allele dosages multiplied by their effect weight (**A**, SUM). The PGS Catalog Calculator also includes PCA-based adjustment methods to normalize PGS mean (**B**, Z_norm1) and mean + variance (**C**, Z_norm2). Population labels represent similarity to super-population labels in a reference panel (e.g. SAS means similar to the South Asian population descriptor in 1000 Genomes).

PGS adjustment methods include comparison to a reference distribution calculated from a similar population, and continuous Principal Component Analysis (PCA)-based adjustments (see Figure 3). The reference populations supported by pgsc_calc include the largest globally representative open access genotypes from the Human Genome Diversity Project and the 1000 Genomes project. Portability enables users to ‘bring code to the data’ on platforms such as Trusted Research Environments, High Performance Computing (HPC) clusters and the cloud, for more equitable application of PGS. In the last 6 months, the PGS Catalog Calculator has been deployed to 10 biobanks to investigate PGS effects on cumulative disease incidence in a diverse cohort of over 1 million participants as part of the INTERnational consortium of integratiVE geNomics prEdiction. An active GitHub community including documentation, issues tracker and discussion forum provides support to any user wishing to reuse scores in the PGS Catalog and calculate PGSs for their own cohorts.

While non-European ancestry data remain low as a proportion of total data, following measures taken by PGS and others to highlight lack of diversity, the availability of non-European ancestry data is now increasing (6).

### Enhancing the DECIPHER platform for clinical variation interpretation

DECIPHER is a web-based platform for sharing phenotype-linked variant data from rare disease patients (7,8). Its dynamic interfaces provide context to enable the use of genetic and phenotypic data by clinical and research genomic medicine communities, for accurate variant interpretation and patient diagnosis. Initially launched in 2004 at the Wellcome Sanger Institute, in 2023 DECIPHER was added to EMBL-EBI’s suite of genetic variation and disease data resources.

DECIPHER provides interactive interfaces for the evaluation of evidence for clinical variant interpretation, based on the American College of Medical Genetics and Genomics (ACMG)/Association for Molecular Pathology sequence variant guidelines (9) and ACMG/ClinGen technical standards for copy number variants (10). DECIPHER also displays summary information and links to ClinGen Variant Curation Expert Recommendations, ClinGen Expert Panel Interpretations and the ACMG recommendations on the reporting of secondary findings.

**Figure 4.**
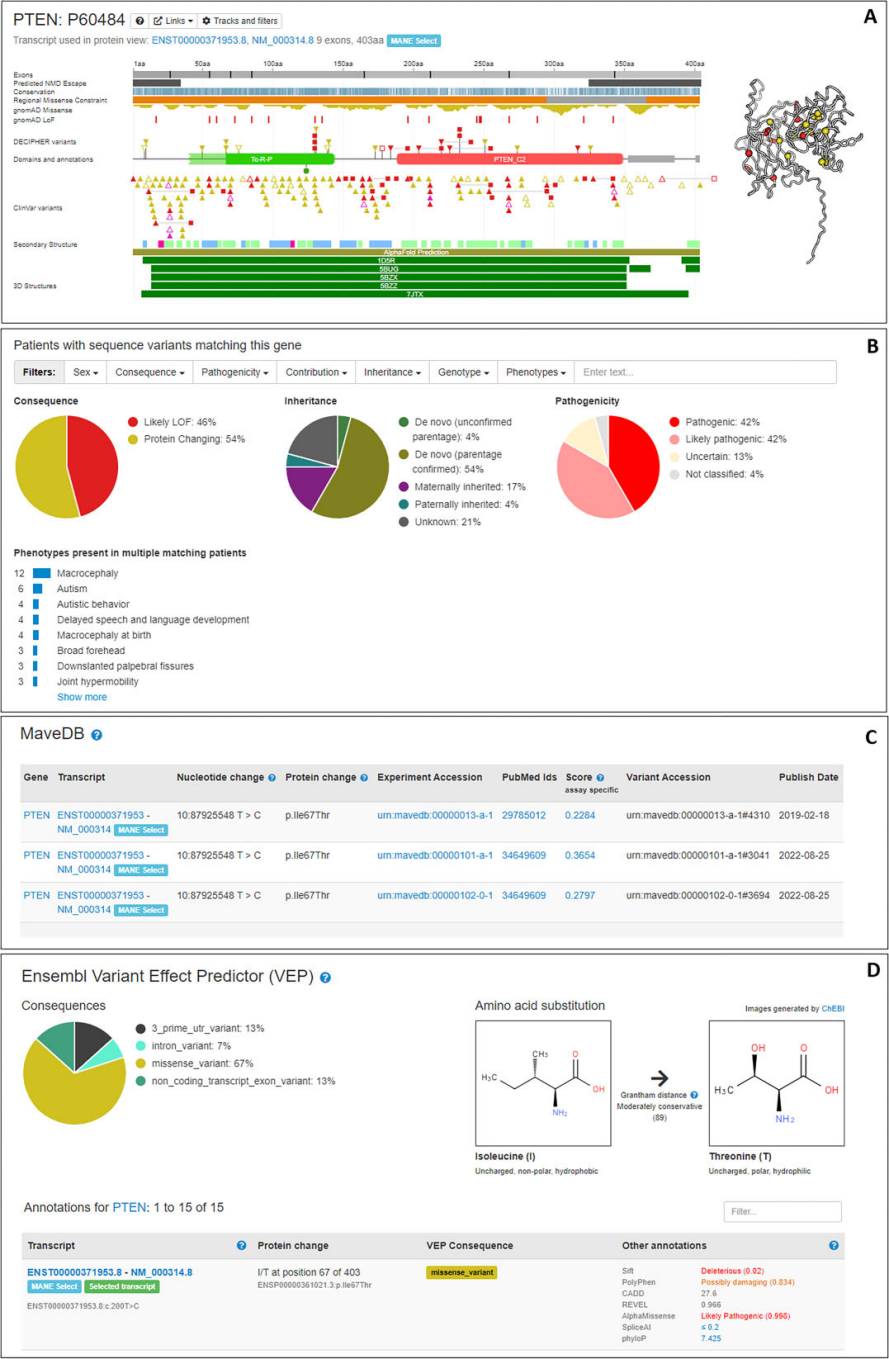
DECIPHER contextualizes genotypic and phenotypic data. Examples shown are for PTEN. (**A**) Protein browser, including AlphaFold predicted 3D structure. (**B**) Matching sequence variant interface. (**C**) MaveDB data. (**D**) Ensembl VEP annotations.

DECIPHER develops intuitive visualizations which contextualize genotypic and phenotypic data, based on datasets describing population, patients, proteins, functional studies and other literature sources (Figure 4). In 2024, functional data from Multiplexed Assays of Variant Effect (MAVEs) deposited in MaveDB (11) were added to DECIPHER. MAVE data provide functional evidence of variant effects, e.g. from deep mutational scanning experiments.

Computational predictions can also be investigated in DECIPHER. Protein-coding variants can be visualized on AlphaFold-predicted 3D structures (12) to investigate potential impact. In 2024, AlphaMissense (13) scores were added to variant displays. AlphaMissense scores categorize missense variants as likely pathogenic or likely benign, providing an indication of the most functionally important parts of a protein. Non-coding variants are increasingly being investigated for impact potential on disease. To aid their interpretation, DECIPHER displays predictive CADD (14) and SpliceAI (15) scores. Finally, we integrated results from UTRannotator (16) which annotates variants in 5′ untranslated regions to predict creation or disruption of upstream open reading frames.

Innovations in available data and recommendations for variant interpretation are evolving. We will continue to integrate these innovations to make the latest research data easily accessible. As establishing disease association is complicated for conditions with incomplete penetrance, one focus in 2025 will be integrating additional case-control datasets to aid variant interpretation for these conditions.

### Developments in the PRIDE database

The PRIDE database is the most popular data repository worldwide for mass spectrometry (MS)-based proteomics data (17).

In 2024, PRIDE CrossLinking launched as a new PRIDE section devoted to MS crosslinking experiments, which are the most mature technique in the interface between proteomics and structural biology. A new infrastructure incorporates open-source third-party software (the xiVIEW tool), enabling improved access and visualization to crosslinking proteomics data, including the exploration of protein–protein interactions and protein complexes. Additionally, cross-references are available to the corresponding structures in PDBe (18) and PDB-Dev (for experimentally generated structures), and AlphaFoldDB (for predicted structures) (19). The approach followed for crosslinking data represents a model to make other types of proteomics data more FAIR.

PRIDE continues to develop large-scale workflows for the reanalysis of public proteomics data. One such workflow, quantms (20), is a newly developed, open-source Nextflow pipeline designed for large-scale reanalysis. It leverages the recently introduced SDRF-Proteomics metadata format (21) and integrates open-source tools like OpenMS (22) to facilitate the analysis of extensive proteomics experiments on cloud or HPC infrastructures. quantms has been utilized to reanalyse over 100 public datasets and 13 000 human samples, resulting in the largest collection of quantified human proteins to date (20).

Finally, the PRIDE Chatbot (23) offers users a new way to interact with EMBL-EBI data resource documentation and search for datasets, using open-source LLM such as Mixtral and llama-2. If embraced by users, LLM-based features can extend to other EMBl-EBI resources in future.

### Expanded scope of the ChEMBL database

The ChEMBL database – celebrating its 15th anniversary in 2024 – hosts small-molecule drug-like compounds and their measured preclinical bioactivity values on defined targets and in various bioassay set-ups. When launched, ChEMBL ingested pre-clinical data from a set of seven core medicinal chemistry journals. This manual data curation from literature will continue and has, over time, been complemented by direct depositions of data, diversifying the range of bioassays hosted.

ChEMBL 34 (released in March 2024) illustrates this diversified scope, featuring completely new data sources as well as some important additions to previously deposited data sources. New bioactivity data from patents provide a focus on underexplored human targets through collaboration with the Illuminating the Druggable Genome project (38). New drug data from the European Medicines Agency (EMA) provides information on EMA drugs licensed prior to 20 January 2023 (excluding vaccines). Seventy one out of the 882 newly added EMA drugs are only authorized by EMA, rather than from other regulatory bodies such as FDA.

### Enhancing the content and access to MGnify Proteins and promoting responsible data reuse

MGnify (39) is dedicated to microbiome-derived sequence analysis. MGnify Proteins is a 2.4M non-redundant set of predicted protein sequences. These sequences are identified as part of the MGnify assembly analysis pipeline and aggregated, dereplicated, assigned an MGYP identifier, clustered at 90% sequence identity and coverage and made available in versioned releases. Cluster representative sequences are annotated with Pfam (40), and have predicted structures associated with the sequences where available. A major refactoring of the underlying database is substantially improving the metadata associated with protein records, and the overall presentation of the data. Each protein sequence now links back to the specific study, sample and contig location where it was identified, to provide genomic context and contextual metadata. MGnify Proteins is available for download via FTP (FASTA and associated flat files). The entire sequence database can be queried via Google Cloud BigQuery for public MGnify datasets, while the representatives can be queried by accession, or as part of a homology search using HMMER, via the new MGnify Proteins site.

With the increasing focus on how marine metagenomics can be a driver for the blue economy, MGnify now includes information about whether a sample falls within a country’s Exclusive Economic Zone (EEZ). This maps sample geolocation metadata to EEZ shapefiles from marineregions.org, linking to potential access and benefit sharing obligations imposed by that country. By making this information visible and accessible for downstream users we aim to support responsible data-reuse. This approach will soon generalize to all environmental samples available in MGnify.

## New developments in knowledgebases

### Drug target prioritization attributes in the Open Targets Platform

Open Targets is a collaboration between academic partners EMBL-EBI and the Wellcome Sanger Institute, and pharmaceutical company partners GSK, Sanofi, Pfizer, Genentech and MSD. The consortium aims to systematically identify and prioritize potential drug targets through an extensive research programme including the development of open source informatics resources for the global community (24). In 2024, Open Targets celebrates its 10 year anniversary.

**Figure 5.**
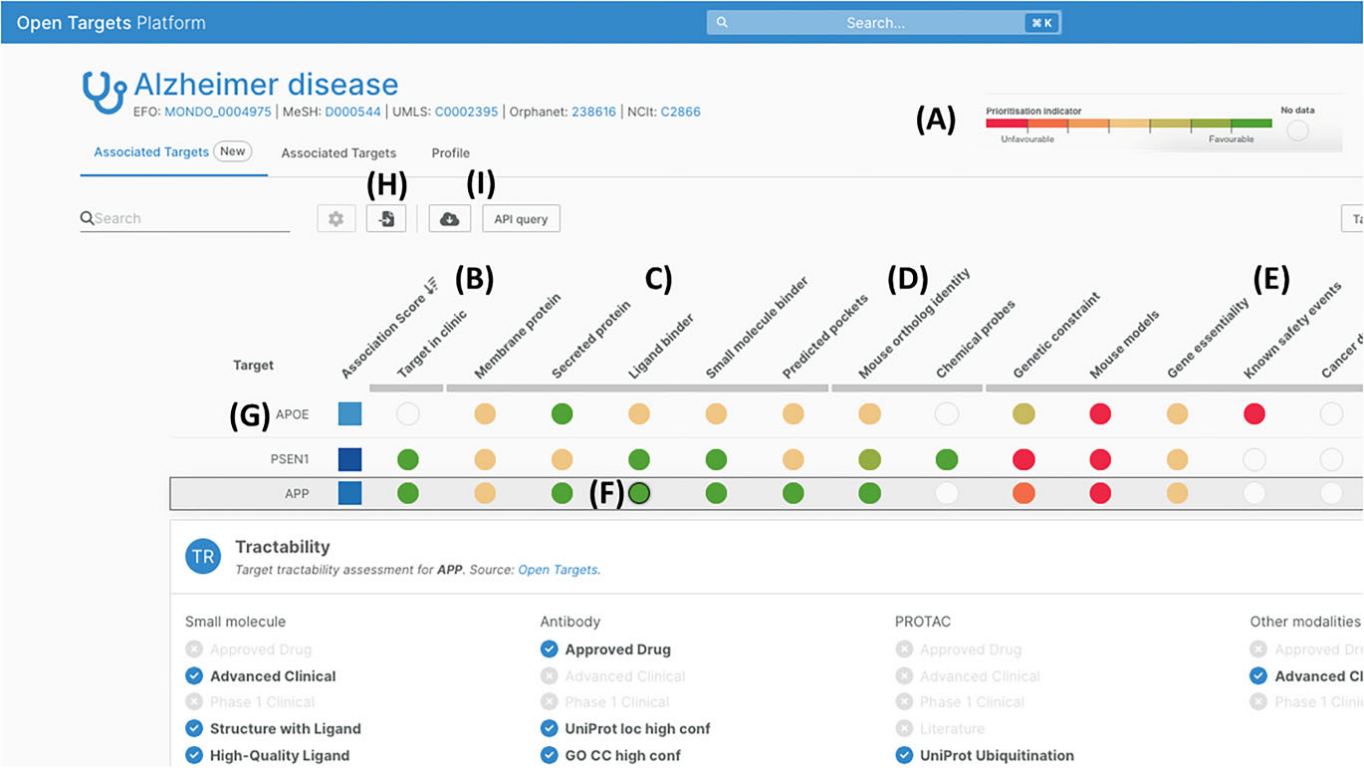
Target attributes page in the Open Targets Platform for prioritizing targets associated with Alzheimer disease.

The Open Targets Platform provides an open source resource to help academic and industry scientists prioritize potential drug targets based upon gene-disease evidence (25). Prioritization is provided as a scored assessment of factors potentially favourable or unfavourable towards the decision to pursue a target for drug discovery (Figure 5A). This extends decision support beyond just disease association evidence, which often produces long lists of candidate targets, through orthogonal progressability factors (24). The scored annotations provided include clinical precedence (Figure 5B), tractability assessments (Figure 5C), doability covering the availability of research tools such as mouse models or probes that could be used for experimental follow-up (Figure 5D) and safety assessments (Figure 5E). The scoring for these attributes was developed as part of an Open Targets funded research project, Target Engine, working closely with industry partners and the Open Targets Platform team at EMBL-EBI.

The interface for target prioritization provides an easy way to explore the underlying evidence for the scored attribute, exemplified for tractability assessments of the APP protein (Figure 5F). A pipeline to provide updated, multi-modality tractability assessments to the platform was developed through an Open Targets funded research project with the EMBL-EBI ChEMBL team (26). Users also have the ability to ‘pin’ targets of interest to the top of the target list or upload a gene list (Figure 5H) to do this automatically. Users can download or share the results via a link, and explore further via the API playground (Figure 5I).

Ongoing work that will enable future feature development in this area is exploring the alignment of direction of effect between disease and association evidence, visualization of groups of disease associated targets based on the biology they are involved in and the novelty of the evidence supporting target-disease associations.

### Integration of clinical, genomic and functional data in CancerModels.Org

Patient-derived cancer models (PDCMs), including patient-derived xenografts (PDXs), organoids and cell lines, are an essential tool in cancer research and precision oncology (27). CancerModels.Org, which launched in February 2023 as a successor of the PDX Finder portal (28), is the largest cancer research platform aggregating clinical, genomic and functional data from PDCMs (29). It unifies and standardizes over 8900 PDCMs and associated data from 51 academic and commercial model provider sources, including frequently mutated genes, diagnoses, drug treatments and sequence data from PDXs, organoids and cell lines (30). CancerModels.Org aims to enable more efficient drug screening, studies of tumour biology and drug resistance and development of personalized medicine.

Users can search for, filter and compare FAIR models and data via a web interface or the REST API, explore molecular data summaries for specific cancer types and connect directly with model providers. CancerModels.Org provides users with links to external resources to explore further. These include the publication platforms PubMed (46) and Europe PMC (53); cancer-specific annotation tools COSMIC (47), CIViC (48), OncoMX (49), OpenCRAVAT (50) and ClinGen (10); and raw data archives EGA (2), ENA (1), dbGAP (51) and GEO (52). A model characterization score, based on the adherence to community-developed standards – the PDX-MI (30) and *in vitro* PDCM Minimal Information standard (in preparation) – lets users evaluate the amount of information available on essential model attributes.

To lower the barriers to model sharing, we exposed our data model as a Metadata dictionary and created a user-facing validation service in collaboration with the Overture team at the Ontario Institute for Cancer Research. Our Validator provides immediate feedback on potential errors in submission spreadsheets, ensuring a standardized validation process and resulting in reduced submission time and a higher proportion of well-annotated models. All models and data are available for exploration through a cBioPortal instance and are integrated into the National Cancer Institute’s Center for Cancer Data Harmonization ecosystem.

CancelModels.Org has successfully piloted model submissions from several national cancer networks, including the Italian Alliance Against Cancer and the Singapore Translational Cancer Consortium. In future years, barriers to data submission will be lowered further using open-source LLMs for model and data acquisition (in collaboration with DeepPhe project) and dedicated instances for national networks and consortia.

### Enhancing scientific data discovery using open-source tools

To help users easily navigate the vast amount of scientific literature, Europe PMC extracts key biological concepts, functions and relations from research publications using text-mining. In 2024, Europe PMC has significantly enhanced its text mining features, using machine learning to improve accuracy, extending the platform to extract data from supplemental files and covering new types of data accessions.

The Europe PMC text mining pipeline is a dictionary-based system that identifies biological terms, such as chemicals or experimental methods, mentioned in journal articles and preprints. These terms are surfaced to readers on content using the SciLite tool (31), and are exposed to programmatic users through the Annotations API. While the current dictionary-based method is effective, it can result in incorrect matches, e.g., identifying a verb ‘bear’ as an organism ‘bear’ and is limited in its coverage i.e. terms that are not part of the dictionary but are true positives will not be extracted. Current state-of-the-art approaches for information extraction can help address these limitations (32). Europe PMC recently introduced a machine learning-based filter, which is integrated alongside the existing system. The filter is mainly used to identify false positive annotations, particularly for complex terms related to genes/proteins, diseases and organisms. The filter algorithm has been developed as an open-source project and can be used to upgrade similar dictionary-based text-mining pipelines. The filter is trained on the openly available Europe PMC gold standard dataset (33), and reduces the number of false positive results that occur with a dictionary-only approach by up to 25%. This improved reliability is essential for biocurators, who rely on highly accurate annotation. These improved annotations are accessible both via the website and the Annotations API.

To enhance data discovery, Europe PMC tracks data citations in life science publications and preprints. More than 1.3 million publications in Europe PMC cite 10.8 million datasets from >50 life science databases. Data DOIs, database accessions and resource names are identified by the same text-mining pipeline described above. In 2024, the BioStudies database and the BIA also started providing DOIs to data submitters.

Data citations can be downloaded using FTP or accessed via Annotations API. Similarly to annotations, this feature is integrated into the Europe PMC website—using the ‘Advanced search’, users can find articles that cite specific datasets, or data types, such as protein structures (34). In 2024 Europe PMC expanded the list of database accession and resource name patterns to include AlphaFold DB (19), BIA (3), BRENDA (41), Cellosaurus (42), Rhea (43) and Silva (44). The extended data citation corpus has been used by ELIXIR to demonstrate the impact of core data resources in the life sciences (35). It also served as a seed file for development of the Open Global Data Citation Corpus, a trusted central aggregate of all data citations (36).

**Figure 6.**
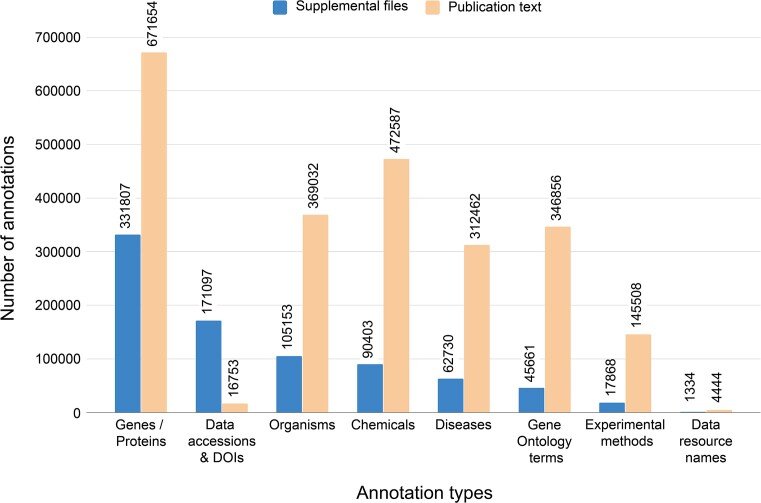
Distribution of biological entity types in text-based supplemental files within BioStudies database (left hand grouped bars) and in corresponding full text publications within Europe PMC (right hand grouped bars). Data accessed via Europe PMC Annotations API on 15 August 2024.

While the publication text summarizes most key data and evidence, many data references are only found within supplementary files (37). This is particularly true for high-throughput analyses that often share complete datasets as supplementary materials. To address this, Europe PMC collaborated with BioStudies to extract data from supplemental files. An open-source text mining REST API was developed, based on the Europe PMC text mining pipeline, capable of processing text-based files to identify biological entities, such as experimental methods, Gene Ontology terms (45), data resources and accessions as well as gene/protein, chemical, organism and disease mentions. BioStudies uses the text-mining API to identify these terms in supplemental files. So far, nearly 800 000 annotations (Figure 6) have been made available via the Annotation API, BioStudies and Europe PMC websites.

This collaborative effort extends the FAIR principles to unlock evidence hidden in supplemental data.

Commitment to open source is an important part of EMBL-EBI’s mission and helps support innovation and discovery—we have made the code and dictionaries required to run the text-mining infrastructure described above openly available, and are already seeing others adapt them for other applications.

## Training

EMBL-EBI’s training programme empowers scientists to make the most of openly accessible data resources and services while developing essential bioinformatics analysis skills. Core principles of FAIR and open data management are embedded in all training activities.

Each year, around 500 scientists attend our live courses, which are offered either in person or virtually, while approximately 500 000 unique IP users access our web-based, on-demand content. We also provide support for trainers, both within EMBL and externally.

Community engagement is central to the ongoing evolution of our programme, including through externally funded collaborations. A revamped partnerships page summarizes these, including our contributions to both emerging and longstanding initiatives such as ELIXIR and the ISCB Education Community.

Web-based on-demand training includes a growing catalogue of online tutorials, curated collections and learning pathways, providing learners with a structured learning approach. A 2024 highlight is the release of AlphaFold online tutorial, co-developed with Google Deepmind, to give researchers an understanding of the fundamental concepts behind AlphaFold2 and how it can be used to enhance research.

Examples of live webinar series from 2024 are Advances in spatial omics and Exploring microbial ecosystems. Series are grouped using a new cover page, providing a series overview, list of competencies gained and links to related live training available for booking. Recordings of all live webinars are made available in our on-demand catalogue.

As well as thematic training, courses often focus on specific EMBL-EBI data resources. The launch of dedicated pages helps users easily find all relevant training material for a specific data resource, e.g. for Ensembl, PDBe and UniProt. These pages also contain links to training material for related EMBL-EBI data resources, making it easier for users to discover and access relevant training for the full range of EMBL-EBI data resources.

## Conclusion

The value of open science and open data has never been clearer, as rapid advances including artificial intelligence applications trained on open data resources are transforming many scientific fields. This was recognized in 2024 by the Nobel committee’s prize in Chemistry for John Jumper and Demis Hassabis, who lead the creation of Alphafold and collaborated with EMBL-EBI to share the resulting protein structure predictions openly via AlphaFold DB (19). These advances are just one example of the value being created by collection and expert curation of reference datasets, co-development of community-driven data standards and guidelines, and provision of tools and training to help scientists use open data to transform their work, whatever and wherever that may be.

## Data Availability

All of the data resources described above are freely available to access at https://www.ebi.ac.uk/services and training is available on https://www.ebi.ac.uk/training/.
